# Diet as a Risk Factor for Early-Onset Colorectal Adenoma and Carcinoma: A Systematic Review

**DOI:** 10.3389/fnut.2022.896330

**Published:** 2022-06-09

**Authors:** Kaitlin L. Carroll, Andrew D. Frugé, Martin J. Heslin, Elizabeth A. Lipke, Michael W. Greene

**Affiliations:** ^1^Department of Nutrition, Dietetics and Hospitality Management, Auburn University, Auburn, AL, United States; ^2^Mitchell Cancer Institute, University of South Alabama, Mobile, AL, United States; ^3^Department of Chemical Engineering, Auburn University, Auburn, AL, United States

**Keywords:** colorectal cancer, early-onset, diet, dietary habits, risk factors

## Abstract

**Background:**

Colorectal cancer in adults 50 years old and younger is increasing in incidence worldwide. Diet may be a modifiable risk factor. The objective of this study was to examine evidence regarding the association between diet and the risk of developing early-onset colorectal cancer (EOCRC) and early-onset colorectal adenomas in young adults.

**Methods:**

PUBMED, Web of Science, and Embase were systematically searched for studies examining dietary intake as a risk factor for EOCRC and early-onset colorectal adenomas. Results were synthesized narratively due to the heterogeneity of the studies.

**Results:**

Of the 415 studies identified, ten met the inclusion criteria. Of these ten studies, four provided data on dietary risk factors for early-onset colorectal adenomas and six provided data on dietary risk factors for EOCRC. The four studies that measured colorectal adenoma occurrence reported an increased incidence with high sugar sweetened beverage intake, a higher pro-inflammatory diet, a higher Western diet score and higher sulfur microbial diet score. A protective effect against early-onset colorectal adenomas was observed in those who had a higher Prudent diet score or higher adherence to other health dietary approaches (Dietary Approaches to Stop Hypertension, Alternative Healthy Eating Index-2010, or the alternative Mediterranean diet). Those who consumed large amounts of deep-fried foods, refined foods, followed a high fat diet, consumed large amounts of sugary drinks and desserts, and had low folate and fiber consumption had a significantly higher occurrence of EOCRC. A protective effect against EOCRC was observed for those who consumed more fruits and vegetables, high amounts of micronutrients and those who adhered to a vegetarian diet.

**Conclusions:**

The results of this study reveal various dietary habits may be risk factors or protective against early-onset colorectal cancer and adenomas. Future research should focus on large prospective cohort studies with long-term follow-up to confirm published results and further examine whether differences in diet quality are associated with EOCRC risk.

## Introduction

Colorectal cancer (CRC) is the 2nd leading cause of cancer deaths and 3rd most commonly diagnosed cancer globally (1). By 2030, the global burden of CRC is expected to increase to more than 2.2 million cases and 1.1 million cancer deaths annually (2). Due to increases in screening, rates among adults aged 50 and older have been decreasing in recent years; however, incidence of cases in those under 50 years old have increased at an alarming rate (3, 4). Early-onset colorectal cancer (EOCRC), defined as colorectal cancer in patients under the age of 50, accounts for ~10% of all newly diagnosed CRC cases (5, 6). The reason for this increase in incidence is unclear.

EOCRC occurs most often in the distal colon and rectum (7). EOCRC is typically discovered at a more advanced stage and more aggressive tumor histology at diagnosis than traditional CRC (6). EOCRC typically arises from neoplasia following the conventional adenoma-carcinoma sequence, therefore, colorectal adenomas are likely common precursors for early onset colorectal cancer (8). A lack of screening and education targeted toward younger adults has contributed to key symptoms and colorectal adenomas going undetected, hindering a recognition and removal process which could likely prevent colorectal cancer (6, 9).

Over 50% of CRC cases diagnosed after age 55 are attributable to modifiable risk factors (10). Known risk factors for traditional CRC include family history, inflammatory bowel disease, low levels of physical activity, cigarette smoking, increased BMI, diabetes and poor dietary habits (11). High consumption of foods that have been shown to increase the risk of CRC include red meat and processed meat, while high intakes of fruits and vegetables, whole grains and dairy products have been associated with a decreased incidence of CRC (12). The risk factors for developing EOCRC are less clear. In countries such as the United States (13), Canada (14), Australia (15) and Japan (16), the rise in early-onset CRC cases occurs mainly in individuals born during or after the 1960s. This strongly indicates that the upward trend in early onset cases could be explained by population-level changes in early-life exposure, such as diet and lifestyle (6, 17). In the US, diet quality declined steadily from 1985 to 2006 (18) and has remained steady since. Worldwide, simple sugar intake has greatly increased in recent decades, mainly due to increased consumption of sugar sweetened beverages (19). Similarly, Western-like dietary habits are increasingly being adopted by non-Western countries. While evidence connecting diet and other modifiable risk factors to traditional CRC is strong, evidence is lacking when it comes to EOCRC due to the expensive cost and difficulties associated with studies that follow young people for an extensive period of time.

It is pertinent to understand the driving factors behind the increase of EOCRC cases, as it typically presents at a more advanced stage, with a greater risk of metastasis and less favorable prognosis (7). Identifying risk factors associated with EOCRC may lead to more effective prevention methods and improve screening efforts for high-risk populations. Even though the traditional risk factors for CRC and their association with precursors for EOCRC and EOCRC risk have been extensively studied, there is a lack of published research articles examining diet alone as a risk factor for EOCRC. Thus, the objective of this systematic review was to examine published research articles on early-onset colorectal cancer and adenomas for risk factors or preventative behaviors related to food choices and dietary habits.

## Methods

### Search Strategy

The methodology follows the PRISMA (Preferred Reporting Items for Systematic Reviews and Meta-Analyses) guidelines. Databases searched include PubMed and Web of Science. The databases were searched using the following key words: risk, early-onset colorectal cancer, diet, young-onset colorectal cancer, risk factors. “And” and “or” were used to combine the keywords listed and to minimize search duplications. There were no language restrictions on the searches. A backwards search was also conducted, i.e., references of accessed studies were evaluated for relevant studies that may have been missed through the database search. A gray literature search of Google was performed. Finally, the Embase database was searched for any articles missed from the above searches. No new articles were found in Embase. Searches were conducted between August 2021 and September 2021.

The primary outcome of this review was the examination of dietary habits that may be risk factors or preventative factors for developing early-onset colorectal cancer and adenomas.

### Inclusion and Exclusion Criteria and Selection Process

The population, interventions, comparators, outcomes and study designs (PICOS) scheme was used for assessing eligibility (Table 1). One reviewer (K.C.) performed an independent quality assessment of the studies to assess eligibility. Eligible studies were peer-reviewed original case-control and cohort studies including men and women diagnosed with early-onset colorectal cancer or colorectal adenoma before age 55. The age eligibility was chosen so that EOCRC studies that did not follow EOCRC being defined as diagnosis at or before age 50 (6) would be included in the current analysis. All cases of colorectal cancer and adenomas were histologically confirmed by a doctor. All studies needed to include a dietary assessment and use the dietary information to examine the association with diet and risk. Excluded study types include reviews, books and book chapters, letters, abstracts, animal studies, cross-sectional studies, and comments on an article.

**Table 1:** The population, interventions, comparators, outcomes and study designs (PICOS) scheme used for assessing eligibility.

**Criteria**	**Description**
Participants	Men and women diagnosed with early-onset colorectal cancer or colorectal adenoma before age 55
Intervention/exposure	Plant-based dietary approaches; Prudent diet; Vegetarian diet; healthy dietary components
Comparison	Western diet; High fat diet; unhealthy dietary components
Outcomes	Colorectal cancer and adenomas histologically confirmed by a doctor
Study design	Case-control and cohort studies

Potential sources were first screened by title and study type to match inclusion criteria. Sources that appeared to match inclusion criteria were then accessed and abstracts were screened. Full-text versions of any remaining articles fitting the inclusion criteria were accessed and assessed.

### Data Collection and Effect Measures

One reviewer (K.C.) collected data from the included reports. All relevant data was compiled into a study characteristics table (Table 2) and independently reviewed by all the investigators.

**Table 2:** Characteristics of included studies (early-onset colorectal adenomas).

**Study**	**Setting and gender**	**Baseline participants**	**Study type**	**Dietary assessment**	**Study details**	**Results**
Joh et al. (20)	United States of America Female	Nurse's Health Study II. 33,106 women included. Inclusion criteria: Women with completed High School FFQ and underwent at least 1 lower gastrointestinal endoscopy between 1999 and 2015. Exclusion Criteria: No lower bowel endoscopy during follow-up, history of cancer except non-melanoma skin cancer, colorectal polyps, Crohn's disease, ulcerative colitis before the return of the HS-FFQ, missing data, implausible energy intake.	Prospective Cohort Study	124 item HS-FFQ for participants diet from age 13–18. Questions asked how often on average a standard portion size of each item was consumed. 9 possible responses.	Primary study outcome: colorectal adenoma Total fructose = sum of free fructose and half sucrose intake. Total glucose = sum of free glucose and half sucrose intake. Added sugar = sugar added during processing/preparation. Western diet pattern = high intake of dessert, sweets, snacks, red and processed meat, refined grains. Prudent diet = high intake of vegetables, fruits, better quality grains, fish, poultry.	Per 1 serving/day higher SSB intake and risk of high risk adenomas: OR = OR = 1.34, CI = 1.01–1.79, *p* = 0.044. (Per 5% of calories) higher total fructose intake during adolescence and risk of high risk adenomas: OR = 1.30, CI = 1.06–1.60, *p* = 0.012. <1.3 fruit servings/day during adolescence and risk: OR = 1.51, CI = 1.26–1.82, *p* < 0.001 (for total fructose) OR = 1.34, CI = 1.12–1.60, *p* = 0.028 (for SSB).
Molmenti et al. (23)	Phoenix, Arizona Male and female	Men and Women recruited from 1990 to 1999 Wheat Bran Fiber and Ursodeoxycholic Acid Phase III chemoprevention trials 1,623 participants included Inclusion criteria: 40–80 years old with 1+ colorectal adenoma removed during colonoscopy evaluation within 6 month period prior to study registration. Exclusion: self-reported inherited syndromes.	Prospective Cohort Study	Dietary intake assessed using Arizona FFQ to evaluate dietary intake over the past few months in the previous year. Energy-adjusted dietary inflammatory index scores derived from FFQ.	Primary study outcome: metachronous colorectal adenoma (characterized as advanced or non-advanced)	Men and women < 50 YO with colorectal adenomas had a higher intake of protein (*p* = 0.03), total fat (*p* < 0.00) monounsaturated fat (*p* = 0.001), polyunsaturated fat (*p* = 0.049), red meat (*p* = 0.001) and more pro-inflammatory diet (*p* < 0.001) than those over 50. < 50 YO red meat consumption > 511 g/week and adenoma risk: OR = 0.84, CI = 0.233–3.021, *p* = 0.79.
Zheng et al. (22)	United States of America Female	Nurse's Health Study II 29,474 women included. Inclusion criteria: Undergone at least 1 lower endoscopy before 2011, younger than 50. Exclusion criteria: diagnosis of colorectal cancer, inflammatory bowel disease, previous history of colorectal polyps, missing data, implausible energy intake	Prospective Cohort Study	FFQ categorized into 40 groups and factor analysis derived scores for either Western or Prudent dietary pattern Derived DASH diet, Alternative Mediterranean diet, Alternative Healthy Eating Index-2010 scores (DASH 8–40; AMED 0–9; AHEI-2010 0–110).	Cases: confirmed newly diagnosed colorectal adenoma. Non-cases: Lower endoscopy with no adenomas. Primary analysis: associations between diet quality and risk of early-onset adenoma overall and according to high vs. low risk. Secondary analysis: association by location/size/histology; evaluated associations according to malignant potential in 2 logistic regressions using same reference group.	Highest quintile for the Western diet and risk of early-onset adenomas: OR = 1.67, CI = 1.18–2.37, *p* = 0.01. Risk and DASH diet score: OR = 0.65, CI = 0.45–0.93, *p* = 0.009, AMED score: OR = 0.55, CI = 0.38–0.79, *p* = 0.007, Prudent score: OR = 0.69, CI = 0.48–0.98, *p* = 0.03, and AHEI-2010: OR = 0.71, CI = 0.51–1.01, *p* = 0.01. Risk of adenomas in the distal colon and rectum for Western Diet: OR = 1.65, CI = 1.14–2.38, *p* = 0.01, Prudent: OR = 0.68, CI = 0.47–0.99, *p* = 0.04, DASH: OR = 0.63, CI = 0.42–0.94, *p* = 0.01, AHEI-2010: OR = 0.71, CI = 0.49–1.03, *p* = 0.02.
Nguyen et al. (21)	United States of America Female	Nurse's Health Study II 30,818 women included. Inclusion criteria: undergone at least 1 lower endoscopy before the end of follow-up and were younger than 50. Exclusion criteria: CRC or IBD before baseline and before each biennial follow-up, prior history of colorectal neoplasia/polyps, implausible energy intake, missing diet intake.	Prospective Cohort Study	FFQ every 4 years from 1991 (130 food items); 1998 gave HS-FFQ (124 food items) Previous study (Nguyen, Ma, Wang) identified 43 different sulfur-metabolizing bacterial species and foods were categorized as such: processed meats, liquor, low-calorie drinks positively associated with enrichment of bacteria; beer, fruit juice, legumes, vegetables, sweets are negatively associated with bacteria.	Primary endpoint was colorectal adenoma or serrated polyp diagnosed before age 50 Cases: had colorectal adenoma Non-cases: no adenoma Scored by summing the intake of putative foods weighed by their regression coefficients. Cumulative average of all sulfur microbial diet scores available from 1991- 2 year questionnaire cycle before most recent endoscopy calculated.	Highest quartile of sulfur microbial diets and risk of early-onset adenomas: OR = 1.13, CI = 1.10–1.56, *p* = 0.02. Association with high sulfur microbial diet and adenomas in the proximal colon: OR = 1.58, CI = 1.17–2.14, *p* = 0.01 and tubulovillous/villous histology: OR = 1.65, CI = 1.12–2.43, *p* = 0.04.

Relevant data included any measures that assessed EOCRC and early-onset adenoma risk with specific foods, dietary habits or specified diets. Additionally, data on alcoholic beverages alone was excluded; however, two studies included alcoholic beverages in their diet score (Alternative Mediterranean and Sulfur Microbial Diet Score), and therefore were not excluded. Data on BMI, weight and obesity levels were not included in this review. Data regarding participant recruitment methods, participant demographics and dietary assessment methods was assessed.

The effect estimates such as odds ratios (OR) and hazard ratios (HR), 95% confidence intervals (CI) and *p*-values (when available) for exposure categories of all dietary factors assessed were extracted. Odds ratios that were adjusted for multi-variables such as age, sex or other risk factors of CRC were the preferred forms of data extracted. All of the extracted data was stored in an electronic table.

### Synthesis of Results

As a result of the significant heterogeneity of the included studies with regard to study design, outcome measures and nutrition assessments, a quantitative synthesis was not possible.

## Results

**Figure 1:**
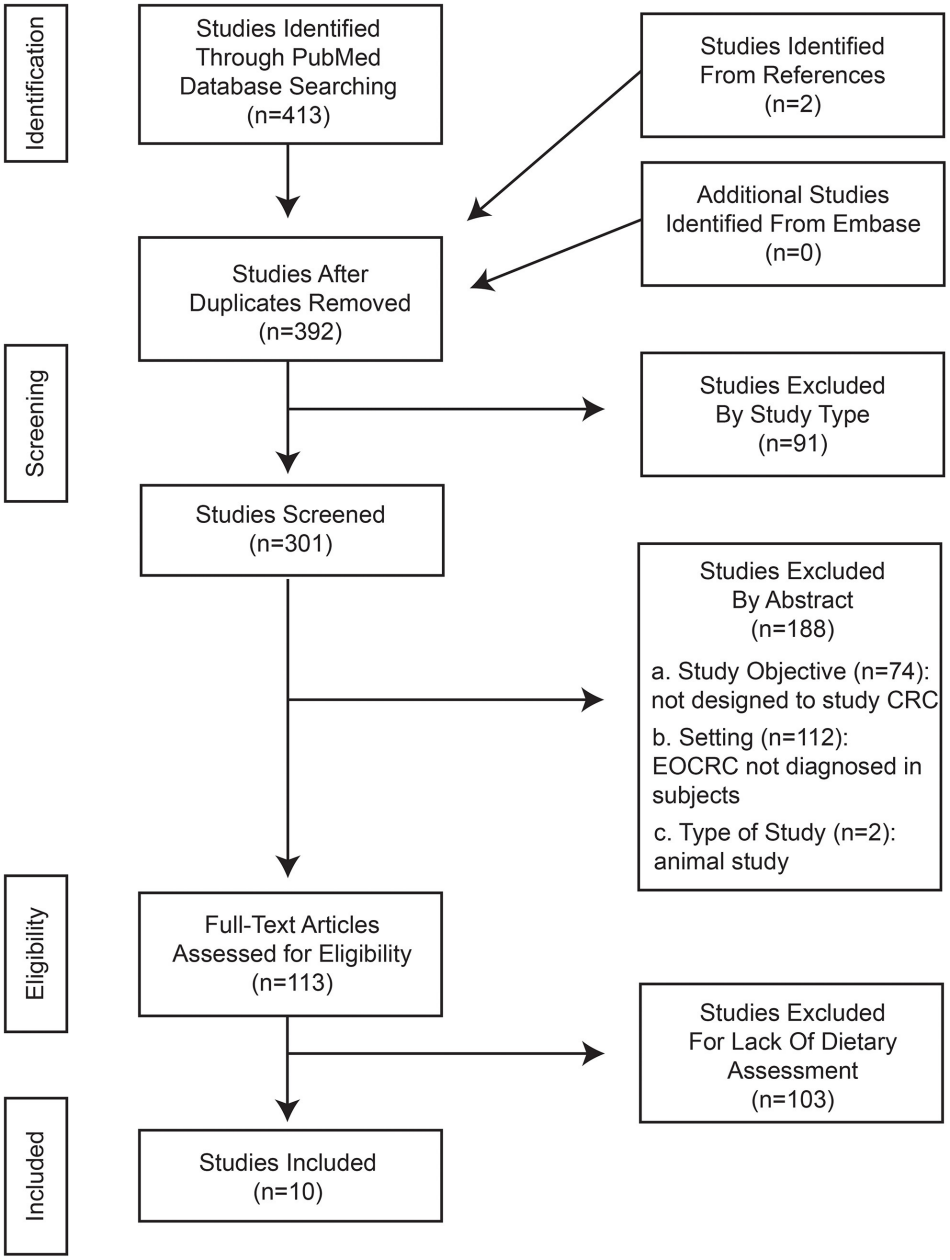
PRISMA flow chart.

In total, 413 studies were identified using the keyword searches. All articles were found on PubMed. Web of Science did not provide any additional articles not already found on PubMed. Two additional studies were identified through backwards searching. After removing duplicates and articles excluded by study type, 301 articles remained for screening. Of these studies, 188 were excluded based on their abstract. From these studies, 113 were assessed for eligibility based on the inclusion and exclusion criteria previously described. One hundred and three of these studies were excluded for lacking inclusion criteria (no dietary assessment). Ultimately, ten articles were included in the final analysis (Figure 1).

### Study Characteristics and Participants

Of the ten studies included, five were prospective cohort studies (20–24) and five were a case-control studies (25–29). Four studies examined the relationship between early onset colorectal adenomas (Table 2) and potential risk factors, including diet (20–23) and six examined risk factors, including diet, for developing early onset colorectal cancer (24–29) (Table 3). The geographic location of the studies were as follows: seven studies used participants in the United States (20–25, 29); two studies used participants from Canada (25, 27); one study included participants from Australia (25); two studies used participants from Asia (25, 28); and two studies used participants in Europe (25, 26). The number of subjects ranged from 74 to 94,217. Ages of the subjects ranged from 19 to 55, apart from two studies which compared early-onset cases to older onset cases (23, 25). All studies used a food frequency questionnaire (FFQ) to assess dietary habits from 1 to 2 years before diagnosis except one, which utilized a high school food frequency questionnaire (HS-FFQ) to assess dietary habits during adolescence and the association with risk (20).

**Table 3:** Characteristics of included studies (early-onset colorectal cancer).

**Study**	**Setting and gender**	**Baseline participants**	**Study type**	**Dietary assessment**	**Study details**	Results*
Yue et al. (24)	United States of America Female	Nurse's Health Study II 94,217 women included. Exclusion criteria: prior diagnosis of cancer (except non-melanoma skin cancer) or inflammatory bowel disease at baseline, those with implausible energy intakes.	Prospective Cohort Study	FFQ self-administered every 4 years since 1991. Prime Diet Quality Score (PDQS) is comprised of 21 food groups. Plant-based scores based on 18 food groups. Empirical dietary index for hyperinsulinemia = weighted sum of 18 food groups.	Primary endpoint = colorectal cancer. Average daily nutrient intake calculated by multiplying the frequency of intake by the nutrient content of each food and summing nutrient values across all foods. Averages computed from all questionnaires up to the start of each 2-year follow-up questionnaire PDQS score: 0–42 Plant-based scores: 18–90.	Plant-based diet indices and EOCRC risk stratified by age: HR = 1.24, CI = 0.74–2.08, *p* = 0.54. PDQS and EOCRC risk stratified by age: HR = 0.90, CI = 0.55–1.50.
Peters et al. (29)	Los Angeles County, California Male	147 case-control pairs. Cases: white males diagnosed with adenocarcinoma of colon or rectum at or before age 45. Controls: matched to cases based on race, sex, date of birth within 5 years, neighborhood of residence, marital status, height, birthplace, religion.	Case-Control Study	All participants given same interview with questionnaire. Subjects asked how often they had eaten several foods over most of their adult life (once a week or less, 2–4x/week, 5+x/week).	Pathology of each case was examined to determine subsite of primary lesion (transverse/ascending colon, sigmoid, right-sided, rectum).	Consumption of deep fried foods > 5x/week and risk for rectal cancer: OR = 4.3, CI = 1.5–12.1, *p* = 0.01; tumors on the right side of the colon: OR = 3.9, CI = 1.4–10.7, *p* = 0.008. Consumption of raw fruits and vegetables and colon cancer risk: *p* = 0.006. A greater consumption of red meat not associated with risk of any subsite.
Khan et al. (28)	Karachi, Pakistan Male and female	74 total colorectal cancer cases (39 female and 35 male). Cases: recruited from surgical oncology unit of Civil Hospital Karachi when colorectal cancer diagnosis confirmed by histopathology examination of biopsy. Controls: Two age and gender matched controls (one sibling, one neighbor; a friend in similar socio-economic situation and same age/gender used if there was an absence).	Case-Control Study	Structured questionnaire divided into 6 parts. Dietary section asked frequency of consumption of rice and rice powders, refined grains and sugars, high fat diet, vegetarian or non-vegetarian.	Vegetarian defined as refrained from eating any kind of meat from animals. Non-vegetarian defined as abstained from any food derived from vegetables. High-fat diet defined as continuous consumption of butter, cheese, whole eggs, nuts, yogurt, etc.	Refined foods and EOCRC risk: OR = 0.01, CI = 0.00–0.04 (unadjusted) A vegetarian diet and EOCRC risk: OR = 0.06, CI = 0.02–0.22 High fat diet and EOCRC risk compared to those who avoided this diet: OR = 1.98, CI-1.13–3.49, *p* = 0.017. (unadjusted)
Chang et al. (27)	Ontario, Canada Male and female	Cases: identified through Ontario Cancer Registry; between 20 and 49 at age of diagnosis and pathologically confirmed incident of invasive colorectal adenocarcinoma (between Jan 2018- May 2019). 175 cases total (58% female). Controls: Ontario residents aged 20–49, no history of CRC, contacted via telephone, 253 total.	Case-Control Study	Self-reported online questionnaire sent via email. Dietary intake assessed via frequency of consumption for specified serving size. FFQ asked about supplement use, consumption of various foods: fruits, vegetables, high-fiber/whole grain, red meat, processed meat, sugary desserts, fast food, canned foods, processed snacks, beverages, sugar substitutes. “Western-like” dietary pattern score calculated.	Dietary assessment was for “2 years ago” from present time of questionnaire. “Western-like” dietary pattern score between 0, 1, 2, 3 assigned for non-beneficial components (red meat, processed meat, sugary drinks, sugary desserts, fast food, processed snacks). Scores reversed for beneficial foods (fruits, vegetables, high fiber/whole grain). Final score calculated by summing up all values. Scores 0–27 (higher = more westernized).	Consumption of 7+ sugary drinks/week and EOCRC risk: OR = 2.99, CI = 1.57–5.68, *p* = 0.002. Consumption of desserts 3–6x/week and risk of EOCRC: OR = 2.28, CI = 1.28–4.04. Consumption of fast food 2+ times/week compared to <1x/week and risk of EOCRC: OR = 1.84, CI = 0.98–3.46, *p* = 0.07. 5+ servings of red meat/week and EOCRC risk: OR = 1.06, CI = 0.56–1.98. Higher Western diet score compared to lower and EOCRC risk: OR = 1.92, CI = 1.01–3.66, *p* = 0.047.
Rosato et al. (26)	Italy (greater, Milan, Pordenone, Forlì, Rome, Latina, Naples) and Switzerland (Swiss Canton of Vaud) Male and female	Cases: histologically confirmed colorectal cancer in major teaching and general hospitals. Controls: admitted to the same hospitals for wide spectrum of reasons. 329 cases and 1,361 controls all ≤ 45 YO were included. 208 colon cancer, 121 rectal cancer. Age range 19–45.	Case-Control Study	Trained interviewer administered questionnaire. Food info based on FFQ and aimed at assessing diet during the 2 years preceding diagnosis or hospital admission (for controls). Asked about 78 foods, food groups or recipes.	Data derived from 3 case-control studies conducted between 1985 and 2009.	EOCRC risk with highest tertile compared to lowest of beta-carotene: OR = 0.52, CI = 0.37–0.72, *p* < 0.001, vitamin C: OR = 0.68, CI = 0.49–0.94, *p* = 0.02, vitamin E: OR = 0.38, CI = 0.26–0.58, *p* < 0.001, and folate: OR = 0.59, CI = 0.40–0.86, *p* = 0.006. EOCRC risk and high consumption of vegetables: OR = 0.40, CI = 0.28–0.56, *p* < 0.001; fruits: OR = 0.75, CI = 0.54–1.02, *p* = 0.073 A greater consumption of red meat and risk: *p* = 0.63.
Archambault et al. (25)	US, Canada, Australia, Asia, Europe Male and female	Participants gathered from 3 large cohort studies: Colon Cancer Family Registry, Colorectal Transdisciplinary study, Genetics and Epidemiology of Colorectal Cancer Consortium. 3,767 cases < 50 YO, 4,049 controls < 50 YO 23,437 cases > 50 YO, 35,311 controls > 50 YO Cases: confirmed CRC with medical record, pathology report or death certificate Controls: identified based on study-specific eligibility and matching criteria.	Case-Control Study	FFQ used to measure dietary factors. Included: fruit intake, vegetable, red meat, processed meat (all servings/day) total calcium, (mg/d) total dietary fiber (g/day), total folate (mcg/day).	Analyses restricted to participants of genetically defined European descent. Exposures assessed before diagnosis by answering questions with mindset of 1–2 years prior to selection. All dietary variables modeled as sex and study-specific quartiles; reference level was category linked to lowest risk based on previously published studies.	Lower folate consumption and EOCRC risk: OR = 1.14, CI = 1.04–1.24, *p* = 0.003 for colon cancer; OR = 1.24, CI = 1.11–1.37, *p* < 0.001 for rectal cancer. Low fiber consumption and risk: OR = 1.14, CI = 1.02–1.27, *p* = 0.02 for colon cancer; OR = 1.30, CI = 1.14–1.48, *p* < 0.001 for rectal cancer. A multivariable model for CRC risk and low folate: OR = 1.08, CI = 0.98–1.18, *p* = 0.11 and fiber consumption OR = 1.11, CI = 1.00–1.23, *p* = 0.06. Low calcium intake and risk: OR = 1.15, CI = 1.05–1.26, *p* = 0.003 for colon; OR = 1.24, CI = 1.11–1.39, *p* < 0.001 for rectum. Multivariable model for low calcium intake: OR = 1.09, CI = 0.99–1.19, *p* = 0.08. Multivariable model high red meat consumption and risk: OR = 1.10, CI = 1.04–1.16, *p* < 0.001.

### Relationship Between Dietary Patterns and Early Onset Colorectal Adenoma Risk

Two prospective cohort studies explored the relationship between dietary patterns and the risk of developing pre-cancerous colorectal adenomas (20, 23). One study investigated if women's sugar sweetened beverage consumption during adolescence had an impact on adenoma risk during adulthood (20). Results revealed a higher sugar sweetened beverage intake (per 1 serving/day) was significantly associated with increased risk of high adenomas (OR = 1.134, CI = 1.01–1.79, *p* = 0.044). Higher total fructose intake (per 5% of calories) during adolescence was associated with an increased risk of high-risk adenomas (OR = 1.30, CI = 1.06–1.60, *p* = 0.012). No significant relationship was observed for artificially sweetened beverages or fruit juice during adolescence and adenoma risk, nor sugar and sugar sweetened beverages during adulthood. The positive associations with sugar intake and adenoma risk were stronger in women who had low fruit intake (<1.3 servings/day) during adolescence than women with high fruit intake. The second study compared dietary patterns of those under 50 years old to those over 50 years old with metachronous colorectal adenomas, defined as sequential adenomas that develop more than 6 months after the initial adenoma, to identify if any foods were significantly associated with a higher risk (23). Compared to adults over 50 years old, men and women under 50 years old who developed colorectal adenomas were more likely to have a high intake of protein (*p* = 0.03), total fat (*p* < 0.00), monounsaturated fat (*p* = 0.001), polyunsaturated fat (*p* = 0.049), red meat (*p* = 0.001) and consume a more pro-inflammatory diet than those over 50 years old (*p* < 0.001). However, a multivariate logistic regression model stratified by age group showed no effect that a high red meat consumption (>511 g/week) was associated with risk of developing metachronous colorectal adenomas for those under 50 years old (OR = 0.84, CI = 0.233–3.021, *p* = 0.79) (23).

### Relationship Between Diet Quality and Early Onset Colorectal Adenoma Risk

Two prospective cohort studies examined the relationship between diet quality and adenoma risk (21, 22), and showed that the overall quality of the diet was inversely associated with risk. Zheng et al. categorized diets into either Western or Prudent, as well as derived scores for the Dietary Approaches to Stop Hypertension Diet (DASH), Alternative Mediterranean diet (AMED) and Alternative Healthy Eating Index-2010 (AHEI-2010) (22). Scores were derived by categorizing FFQ data into groups and performing a factor analysis. Those in the highest quintile for the Western diet has an increased risk of early-onset adenomas with a higher malignant potential, whereas the DASH, AMED, Prudent and AHEI-2010 showed significant inverse associations for risk of developing early-onset high-risk adenomas (OR = 1.67, CI = 1.18–2.37, *p* = 0.01; OR = 0.65, CI = 0.45–0.93, *p* = 0.009; OR = 0.55, CI = 0.38–0.79, *p* = 0.007; OR = 0.69, CI = 0.48–0.98, *p* = 0.03, OR = 0.71, CI = 0.51–1.01, *p* = 0.01, respectively) (22). The Western, Prudent, DASH and AHEI-2010 diets showed significant associations for the risk of advanced adenomas in the distal colon and rectum (OR = 1.65, CI = 1.14–2.38, *p* = 0.01; OR = 0.68, CI = 0.47–0.99, *p* = 0.04; OR = 0.63, CI = 0.42–0.94, *p* = 0.01; OR = 0.71, CI = 0.49–1.03, *p* = 0.02, respectively).

Through the use of scoring diets for their potential to enrich sulfur-metabolizing bacteria, Nguyen et al. found women in the highest quartile of sulfur microbial diets (most enrichment potential) had an increased risk of early-onset adenomas (OR = 1.13, CI = 1.10–1.56, *p* = 0.02) (21). Adenomas in the proximal colon and those characterized with a tubulovillous or villous histology showed a larger association with adherence to a high sulfur microbial diet (OR = 1.58, CI = 1.17–2.14, *p* = 0.01; OR = 1.65, CI = 1.12–2.43, *p* = 0.04, respectively) (21).

### Association Between Micronutrients and Early Onset Colorectal Cancer Risk

The relationship between micronutrient intake and the risk of developing EOCRC was explored in two studies (25, 26). In a case-control study, Rosato et al. found those in the highest tertile of consumption of beta-carotene, vitamin C, vitamin E and folate had a protective effect against EOCRC risk compared to those in the lowest tertile (OR = 0.52, CI = 0.37–0.72, *p* < 0.001; OR = 0.68, CI = 0.49–0.94, *p* = 0.02; OR = 0.38, CI = 0.26–0.58, *p* < 0.001; OR = 0.59, CI = 0.40–0.86, *p* = 0.006, respectively) (26). Archambault et al. found that lower folate consumption was linked to a greater risk of EOCRC by subsite (OR = 1.14, CI = 1.04–1.24, *p* = 0.003 for colon cancer, OR = 1.24, CI = 1.11–1.37, *p* < 0.001 for rectal cancer), as well as lower fiber consumption (OR = 1.14, CI = 1.02–1.27, *p* = 0.02 for colon cancer, OR = 1.30, CI = 1.14–1.48, *p* < 0.001 for rectal cancer) (25). However, a multivariable model adjusted for age, sex, study, family history and total energy consumption failed to show significance for low folate and fiber consumption, but trended toward an increased colorectal cancer risk (*p* = 0.11 and *p* = 0.06, respectively) (25). Archambault observed an increased risk of colon cancer and rectal cancer for low calcium intake (OR = 1.15, CI = 1.05–1.26, *p* = 0.003; OR = 1.24, CI = 1.11–1.39, *p* < 0.001, respectively) and a trend toward higher risk was observed in a multivariable model (OR = 1.09, CI = 0.99–1.19, *p* = 0.08) (25).

### Relationship Between Dietary Patterns and Early Onset Colorectal Cancer Risk

Four studies explored how different dietary patterns were associated with EOCRC risk (26–29). In a case control study, consumption of deep fried foods > 5x/week was associated with an elevated risk for rectal cancer and tumors on the right side of the colon (OR = 4.3, CI = 1.5–12.1, *p* = 0.01; OR = 3.9, CI = 1.4–10.7, *p* = 0.008, respectively) (29). Consumption of refined foods including corn flakes, pastas, noodles, pizza and refined sugars, appeared protective against EOCRC as reported in the Khan et al. case-control study (OR = 0.01, CI = 0.00–0.04) (28). Greater consumption of sugary drinks (7+ drinks/week) and desserts (3–6x/week) was associated with an elevated risk of EOCRC (OR = 2.99, CI = 1.57–5.68, *p* = 0.002; OR = 2.28, CI = 1.28–4.04) (27). Consumption of fast food two or more times/week showed a suggestive, but not significant, association with increased risk of EOCRC compared to consumption <1x/week (OR = 1.84, CI = 0.98–3.46, *p* = 0.07) (27). Two studies categorized diets according to their adherence to a plant-based diet. Khan et al. found a vegetarian diet contributed a protective affect against EOCRC (OR = 0.06, CI = 0.02–0.22) (28); however, a plant-based diet was not associated with CRC (HR = 1.24, CI = 0.72–2.16, *p* = 0.54) in a study with only female participants (24). Two studies observed a protective effect against EOCRC for increased citrus fruit and vegetable consumption (*p* = 0.006) (29) (Vegetables: OR = 0.40, CI = 0.28–0.56, *p* < 0.001, Citrus Fruits: OR = 0.61, CI = 0.45–0.84, *p* = 0.002) (26). A greater consumption of red meat was associated with an increased risk of EOCRC in a study with only female participants (OR = 1.10, CI = 1.04–1.16, *p* < 0.001) (25); however, three studies that included male participants showed no association (26, 27, 29).

### Relationship Between Diet Quality and Early Onset Colorectal Cancer Risk

Two studies examined the relationship between diet quality and EOCRC risk (27, 28) and showed that the overall quality of the diet was inversely associated with the risk of developing CRC at a young age. Categorizing and scoring diets according to their similarities to a Western diet showed an elevated risk of developing EOCRC for those with the higher Western diet score compared to lower (OR = 1.92, CI = 1.01–3.66, *p* = 0.047) (27). Additionally, a high fat diet was significantly associated with a higher chance of developing EOCRC compared to those who did not consume this diet (OR = 1.98, CI = 1.13–3.49, *p* = 0.017) (28).

## Discussion

Currently, there is extensive evidence pointing to an association between certain dietary factors and risk for developing older-onset colorectal cancer. However, there is much less known about the modifiable risk factors for developing early-onset colorectal cancer. As such, it has been difficult to identify groups of people at risk for EOCRC based on modifiable risk factors, such as dietary patterns, and flag them for early screening or other preventative actions. Unlike other published systematic reviews, this systematic review focused solely on diet as a risk factor for developing or preventing early-onset colorectal cancer and colorectal adenomas.

Approximately 85% of early-onset colorectal cancer cases develop through the conventional adenoma-carcinoma sequence (20). Therefore, it was important to investigate dietary risk factors for both potentially precancerous early-onset colorectal adenomas and EOCRC. One of the four studies that analyzed risk factors for colorectal adenomas focused on sugar sweetened beverage (SSB) consumption both during high school and adult life (20). It was shown that a high SSB and fructose consumption during high school significantly increased the risk of developing colorectal adenomas before the age of 50. Additionally, women who consumed less fruit during adolescence were shown to have stronger associations for adenoma risk than those who consumed more. Fruits and liquid fructose have different intestinal release rates due to the fiber content and other cellular components of whole fruits (20). Liquid fructose is rapidly digested, and large amounts of fructose at one time can exceed small intestine uptake capacity and overflow to the colon (20). In addition to the study looking at adenomas, one study found similar results for SSB consumption and EOCRC risk. A higher consumption of sugar sweetened beverages was significantly associated with an increased risk of EOCRC (27). It is believed an overall unhealthy diet combined with excessive sugar intake may exacerbate chronic insulin release, thereby promoting colorectal carcinogenesis. High fructose corn syrup, the main sweetener in beverages since the 1980s, has negative impacts on insulin sensitivity and the gut microbiota, which may play a role in EOCRC etiology (27).

One study investigated how a diet which promotes sulfur-metabolizing bacteria influences early-onset adenoma risk. It was found that a high sulfur microbial diet score significantly increased the risk of developing a colorectal adenoma before age 50 (21). A higher score indicated that a participant consumed more foods associated with the enrichment of sulfur-metabolizing microbes in the gut, which may lead to a higher production of pro-carcinogenic hydrogen sulfide (21). Processed meats were included in the category of foods positively associated with the enrichment of sulfur-metabolizing bacteria. Plant-based sulfur sources are distinct from animal-based sources because they are composed of primarily cancer protective compounds, such as glucosinolate, and are negatively associated with the enrichment of the bacteria (21).

Red meat and processed meat are well documented risk factors for colorectal cancer (23) due to their genotoxic effects (30). Despite this, red meat consumption was not shown to be a risk factor for early-onset colorectal adenomas and results were mixed for EOCRC. It was speculated that the lack of significance may have been due to the fact that individuals under 50 years old have experienced a shorter duration of meat exposure, which may not be long enough to produce a significant effect (23). Three large cohort studies agree that red meat consumption may not impact adenoma development until later in life (23, 31, 32). It has not yet been confirmed at what stage of carcinogenesis red meat plays its largest role. However, processed, barbequed and cured meats did increase the risk of EOCRC (27, 29). This is consistent with the hypothesis that N-nitroso compound formation increases CRC risk (29), and that N-nitroso compounds are highly genotoxic (33). Existing evidence is in line with the findings that processed meat is directly associated to EOCRC risk (26, 34).

It has been well established in Westernized countries but also globally over the past several decades that dietary patterns in younger generations are shifting toward more unhealthy diets, such as a Western diet (25). Diets are becoming increasingly heavy in meat, fats, oils, added sugars and sweeteners while vegetables, fruits and whole grain consumption is decreasing (22, 25). The Western diet is characterized by low-fiber, high fat and high sugar, and is known to be more pro-inflammatory than it is anti-inflammatory (27). In the studies reviewed, a high Western diet score was associated with an increased risk of adenoma development, specifically those in the distal colon and rectum (22). A recent cohort study found that a Western diet induces both inflammation and gut dysbiosis, potentially explaining its role in increasing early onset colorectal adenoma risk (27). Consistent with the studies on adenoma risk, a Western-like diet was related to an increased risk of EOCRC (27). A Western diet is more strongly associated with tumors of molecular subtypes common in EOCRC (22). These molecular subtypes are more likely to originate through the conventional adenoma-carcinoma sequence (21), therefore it is not surprising that a Western diet was consistent with an increased risk of both EOCRC and adenomas. Western diets have also been shown to be associated with traditional CRC (22). The results of a Western diet on EOCRC risk suggest overall poor diet quality may also be a risk factor for early-onset CRC (27), which is currently considered a putative risk factor for traditional CRC.

In line with the findings that poor diet quality is a risk factor for EOCRC are studies reporting that a high-fat diet was associated with an increased risk for EOCRC (28), and common among those who developed adenomas before age 50 (23). This is consistent with data that found an increased risk of EOCRC to be associated with a high intake of processed foods, as these foods are often high in fat (29). Unhealthy diets, characterized by high fat and processed food consumption, are linked to obesity which has been shown to be associated with the risk of EOCRC in previous studies (22, 35). However, direct evidence for the effect of consuming a high-fat diet on EOCRC risk is needed.

Healthy diets, as indicated by recommendation based dietary indices, have been shown to be associated with a lower risk of traditional CRC (24). Adherence to the Prudent diet, alternative Mediterranean diet, AHEI-2010 and DASH diet, were shown to have an inverse association with early onset colorectal adenoma risk in both the distal colon and rectum. The DASH diet is characterized by being high in low fat dairy, which is a good source of calcium. High calcium intake has been shown to be inversely related with distal colon cancer, potentially because of its capability to reduce cell proliferation and promote cell differentiation and apoptosis (22). This is in agreement with the two studies included in this review which found low calcium intakes increased EOCRC risk (25). The protective effect of calcium is consistent with evidence for traditional CRC, signifying calcium may have implications for chemoprevention research (27).

It is important to note alcohol consumption was considered when scoring both the sulfur microbial diet and the alternative Mediterranean diet (21, 22). This review excluded most data related to alcohol consumption; however, the intake of these beverages was not able to be separated out of the published results. It cannot be ruled out that the significance seen in these studies' results may have been influenced by the alcoholic beverage consumption, a known risk factor for CRC (36). The sulfur microbial diet score was influenced by liquor and beer intake. Liquor was considered one of the food groups positively associated with the enrichment of sulfur-metabolizing bacteria. Beer was included in the group of foods negatively associated with the enrichment of sulfur-metabolizing bacteria. A higher sulfur microbial diet score indicates one's diet is more enriched for sulfur-metabolizing bacteria, which could enhance the production of pro-carcinogenic compounds (21). Therefore, liquor was considered to be more harmful when it comes to enhancing the risk of CRC and adenomas, and beer may be more protective. Zheng et al. measured alcohol consumption when scoring diets according to the AMED score (22). A traditional Mediterranean diet includes moderate consumption of ethanol, typically in the form of wine (37). Resveratrol, found in the skin and seeds of grapes, is one of multiple polyphenolic compounds found in wine. Polyphenolic content is higher in red wines but varies by the grape varietal and the vinification process (38). Resveratrol's proposed cancer protecting properties include defending against reactive oxidative species induced damage and repressing platelet aggregation (39). Resveratrol in combination with other polyphenolic compounds is believed to be useful in advanced stages of cancer through its ability to deregulate many pathways affecting cancer cell growth and oncogenic signaling (40). Additionally, resveratrol has been shown to have beneficial effects for tumor prevention (41, 42). A greater adherence to a Mediterranean diet has been linked to lower levels of cancer mortality (43). Previously published studies have demonstrated mixed results when it comes to alcohol consumption and traditional CRC. A retrospective analysis performed by Crockett et al. found that moderate intake of wine was inversely associated with CRC (44). However, multiple meta-analyses have found that alcohol increases CRC risk in a linear dose-related manner (45–47). According to the study included in this review, a higher AMED score was associated with a lower risk of early-onset colorectal adenomas, although the amount of alcohol consumed by participants was not listed (22).

Other micronutrients shown to lower the risk for EOCRC include beta-carotene, vitamin, C, vitamin E and folate (26). A second study found that low folate and fiber intake increased the risk for developing EOCRC (25). It was unsurprising high intakes of citrus fruits and vegetables also appeared protective against EOCRC because they are high in fiber and micronutrients, like the ones described previously (26, 29). The positive association between fruit and vegetable intake and EOCRC risk is in line with existing evidence (26). The anti-inflammatory compounds found in various fruits and vegetables may help modulate cancer cell proliferation and apoptosis (28). One study found the consumption of fresh fruit and raw vegetables to be protective in the colon, but not the rectum, which is in agreement with previous case-control studies (29, 48). Despite the positive results from fruit and vegetable consumption, vegetarian diets produced mixed results. One study found a vegetarian diet to be protective, whereas another saw no effect (24, 28). It is possible that the plant-based diet indices used to measure vegetarian diets in one study is tailored more toward critical factors for heart disease, and therefore may be less important for cancer (24).

Many of the studies examined colorectal cancer and adenoma by subsite. The subdivisions of the colorectal area have different blood supplies, patterns of motility and physiological function (29). It has been hypothesized that the specific mechanisms of carcinogenesis parallel the physiological differences (29). This may explain why different areas of the colorectal region were affected by different dietary habits. Because EOCRC typically occurs in the distal colon and rectum, it may be more important to focus on the dietary habits which exhibit risk factors for cancers occurring in these regions. From the studies investigated in this review, the Western diet was associated with an increase in colorectal adenoma risk in the distal and rectal regions (22). The Prudent, DASH and AHEI-2010 diets were protective against adenoma risk in distal and rectal region (22). Low folate, fiber and calcium intakes were related to a higher risk of early-onset rectal cancer, as well as high levels of fast food consumption (25, 29).

Listed in Table 4 are the covariates adjusted for in the included studies. Half of the studies in this review included BMI as a covariate adjustment. BMI was considered a potential confounder due to the link between obesity and traditional CRC, as well as data suggesting obesity is significantly associated with increased EOCRC risk (10, 49). The five studies which did not adjust for obesity cannot rule out the possibility that participants BMI may have impacted their findings. It has been hypothesized that obesity is linked to EOCRC cases because the increasing prevalence of obesity parallels the increasing incidence of EOCRC (49). Obesity has been shown to be associated with earlier onset of metabolic conditions and diseases such as insulin resistance, type 2 diabetes, high blood pressure and dyslipidemia (50). These metabolic conditions may be underlying mechanisms for colorectal neoplasia. The time of BMI measurement relative to disease diagnosis is important when studying EOCRC, as weight loss is a common symptom (6). Therefore, BMI measured at the time of diagnosis may not be as relevant to BMI before diagnosis (49).

**Table 4:** Data adjustments for included studies.

**Study**	**Adjusted for**
Joh et al. (20)	3 multivariable models with adjustments for potential confounders. Model 1 adjusted for age, time period of endoscopy, time since most recent endoscopy, number of endoscopies, reason for endoscopy. Model 2 additionally adjusted for family history of CRC, menopausal status/menopausal hormone use, current aspirin use greater than or equal to 2x/week, history of type 2 diabetes, adult height, body mass index (BMI) (at age 18 and current), smoking (adolescent and current), alcohol consumption (18–22 years and current), physical activity (adolescent and current). Model 3 additionally adjusted for adolescent and adult intake of total calories, total calcium, vitamin D, total folate, fiber, fruits, vegetables and dairy, currently total red meat intake, western dietary pattern score during adolescence, corresponding adult variables to adolescent exposure variables.
Molmenti et al. (23)	Calcium, energy, protein, total fat, saturated fat and supplemental folate intake were rescaled by a factor of 100 to provide large enough coefficient estimation to reasonable capture the change in each variable and its effect of metachronous adenomas. Odds rations and 95% CI adjusted for waist circumference, gender, energy and trial arm.
Zheng et al. (22)	Age-adjusted models controlled for age, total caloric intake, time period of endoscopy, number of reported endoscopies, time in years since most recent endoscopy, reason for current endoscopy. Multivariable models additionally adjusted for height, BMI, history of CRC in first-degree relative, menopausal status, menopausal hormone use, history of type 2 diabetes, pack-years of smoking, physical activity, current use of multivitamin, regular use of aspirin or non-steroidal anti-inflammatory drugs DASH diet further adjusted for alcohol intake.
Nguyen et al. (21)	Covariates adjusted for included age (5-year intervals), time period (2-year intervals), first degree family history of CRC, height, BMI, menopausal status, menopausal hormone use, personal history of type 2 diabetes, pack-years smoking, physical activity, current use of multivitamin, regular use of aspirin or non-steroidal anti-inflammatory drugs, number of reported endoscopies, time in years since most recent endoscopy, reason for most recent endoscopy, total caloric intake (quartiles). For analyses considering high school diet, covariates most proximate to the exposure used. High school sulfur microbial diet scores calculated without alcohol (primary), or assuming alcohol consumption was all beer, all liquor, or spilt between both equally.
Yue et al. (24)	Models stratified by age and follow-up cycle Models adjusted for energy intake (kcal/day) Multivariable models additionally adjusted for total alcohol consumption, height, race, family history of CRC, history of diabetes, smoking pack-years, regular use of aspirin or non-steroidal anti-inflammatory drugs, multivitamin use, menopausal status and hormone use, history of lower endoscopy within the past 10 years. Five dietary indices further adjusted for BMI and physical activity.
Peters et al. (29)	Adjusted for age and education.
Khan et al. (28)	Univariable odds ratios were unadjusted Multivariable odds ratios were adjusted for all independent variables.
Chang et al. (27)	Multivariable models adjusted for covariates that included age, sex, family history of CRC, aspirin/non-steroidal anti-inflammatory drug use, smoking, physical activity, BMI, alcohol consumption, red/processed meat intake, fruit and vegetable intake, high-fiber food intake, calcium supplement use.
Rosato et al. (26)	Unconditional multiple logistics regression models included terms for age, sex, center, study, year of interview, education, family history of CRC, alcohol drinking. The analysis of dietary items models further included terms for total energy intake using the residual method.
Archambault et al. (25)	Adjusted for age, sex, family history, study, total energy consumption.

Of the ten included studies, four exclusively included women (20–22, 24), four were over 50% women (25–28), one exclusively included males (29), and one was predominantly men (62% of participants under 50 years old and 68% of participants over 50 years old were male) (23). EOCRC differs from older-onset CRC in that it affects men and women equally; however, it has not been determined if they also share the same risk factors (36). Due to the heterogeneity of the studies and their range of dietary measures, it was difficult to determine if specific diet components or approaches affected men and women's risk differently. A study with 62% male participants (23) and two studies with 100% female participants (22, 27) both found a pro-inflammatory diet to increase the risk of EOCRC and adenomas. Furthermore, red meat's effect on EOCRC and adenoma risk was not statistically significant when comparing results from men and women (23, 25, 27, 29). When analyzing the demographics of the included studies', nine out of the ten studies included mostly white males and females (20–27, 29), and the tenth included only Pakistani participants (28). The studies with mostly white participants consistently found pro-inflammatory diets, such as the Western diet, and unhealthy foods including SSB, fast food and desserts to have a negative impact of EOCRC and early-onset adenoma risk (20, 22, 23, 27, 29). Healthy dietary choices such as fruits, vegetables and consuming high levels of various micronutrients appeared to be protective against EOCRC and adenoma risk in the studies with white participants (24–26, 29). When comparing the two predominant demographics of the included studies, refined foods and high fat foods increased EOCRC and adenoma risk, and a more plant-focused diet was protective against EOCRC and adenoma risk in both demographics (23, 26–29). The lack of diversity in the studies' participants limited the generalizability of the results, making it difficult to determine if similar dietary habits would affect other races in a comparable manner.

The strengths of this systematic review include reporting according to the PRISMA guidelines. Several limitations exist as well. While this review provides a summary of the most current evidence regarding diet and early-onset colorectal cancer, there were a limited number of studies included, and a meta-analysis could not be conducted due to the heterogeneity of the studies. A lack of dietary assessment of patients with EOCRC is a limitation of the published research. The age range of included participants is a limitation of this review. Currently, there is no clear or widely accepted consensus for a definition of age groups for EOCRC (51). This study intended to adhere to the definition of early-onset as before screening age, i.e., < 50 years old, however; it was difficult due to the limited number of published studies. The mean age of cases in one study was 52.2, with 76.5% of cases being diagnosed before age 55 (20). The average age of cases in a second study was 41.47, with the standard deviation of ages from the mean was 15.54, therefore adults up to the age of 57 were included (28). Long follow-up studies with young individuals are cost-prohibitive; therefore there is a lack of diverse populations studied for EOCRC and adenomas. Four of the included studies utilized the NHSII cohort for their data, limiting the diversity of the populations studied (20–22, 24). Many of the included studies share similar limitations. While FFQs are commonly used in nutrition research, they still are associated with recall bias, misclassification and measurement error. Though multiple countries were included across the studies, most participants were white and often female, reducing the generalizability of these results. It appears EOCRC is similar between males and females, therefore the predominant inclusion of females is not a limitation of the results (7, 52, 53). The possibility of residual confounding factors cannot be ruled out in the observational studies due to the nature of the studies.

The rising incidence in early-onset colorectal cancer cases makes identifying risk factors imperative. More detailed studies on the differences in dietary index and CRC risk by anatomic sites in young adults are needed. Large prospective cohort studies with long follow-ups are needed to confirm results and to help screen for strategies that can be targeted at this sub-population. Elucidating modifiable risk factors for early-onset colorectal cancer, such as diet, is necessary to help slow the rising incidence and preserve life years for the population.

## Conclusions

This review demonstrated that various dietary habits, some of which are already confirmed risk factors for traditional colorectal cancer, may be risk factors or protective against early-onset colorectal cancer and adenomas. These results should be interpreted with caution, as a meta-analysis was not able to be conducted and the generalizability of the results is limited to the populations studied. The lack of knowledge on EOCRC risk factors make the associations identified in this review helpful starting points for dietary habits to be examined in future studies. By identifying modifiable risk factors for EOCRC, such as the dietary choices analyzed in this review, high-risk populations can be targeted for early intervention and prevention methods.

## Data Availability Statement

The original contributions presented in the study are included in the article, further inquiries can be directed to the corresponding author.

## Author Contributions

MG conceived of the work, contributed to the writing, and offered critical comments. KC performed data analysis and wrote the first draft of the manuscript. AF, MH, and EL provided critical revisions to the content. All authors contributed to the article and approved the submitted version.

## Funding

This material is based upon work that was supported by the National Institute of Food and Agriculture, U.S. Department of Agriculture, Hatch/Multistate project Accession ALA044-1-18037 to MG. Research reported in this publication was supported by the National Center for Advancing Translational Research of the National Institutes of Health under award number UL1TR003096 to MG and EL.

## Conflict of Interest

The authors declare that the research was conducted in the absence of any commercial or financial relationships that could be construed as a potential conflict of interest.

## Publisher's Note

All claims expressed in this article are solely those of the authors and do not necessarily represent those of their affiliated organizations, or those of the publisher, the editors and the reviewers. Any product that may be evaluated in this article, or claim that may be made by its manufacturer, is not guaranteed or endorsed by the publisher.
